# Polymeric versus lipid nanocapsules for miconazole nitrate enhanced topical delivery: *in vitro* and *ex vivo* evaluation

**DOI:** 10.1080/10717544.2022.2026535

**Published:** 2022-01-17

**Authors:** Rania S. Abdel-Rashid, Doaa A. Helal, Ahmed Adel Alaa-Eldin, Raghda Abdel-Monem

**Affiliations:** a Department of Pharmaceutics and Industrial Pharmacy, Faculty of Pharmacy, Helwan University, Ain Helwan, Cairo, Egypt; b Department of Pharmaceutics, Faculty of Pharmacy, Fayoum University, Faiyum, Egypt

**Keywords:** Miconazole nitrate, polymeric nanocapsules, polycaprolactone, lipid core nanocapsules, permeation

## Abstract

Nanocapsules can be equated to other nanovesicular systems in which a drug is entrapped in a void containing liquid core surrounded by a coat. The objective of the present study was to investigate the potential of polymeric and lipid nanocapsules (LNCs) as innovative carrier systems for miconazole nitrate (MN) topical delivery. Polymeric nanocapsules and LNCs were prepared using emulsification/nanoprecipitation technique where the effect of poly(ε-caprolactone (PCL) and lipid matrix concentrations with respect to MN were assessed. The resulted nanocapsules were examined for their average particle size, zeta potential, %EE, and *in vitro* drug release. Optimum formulation in both polymeric and lipidic nanocapsules was further subjected to anti-fungal activity and *ex vivo* permeation tests. Based on the previous results, nanoencapsulation strategy into polymeric and LNCs created formulations of MN with slow biphasic release, high %EE, and improved stability, representing a good approach for the delivery of MN. PNCs were best fitted to Higuchi’s diffusion while LNCs followed Baker and Lonsdale model in release kinetics. The encapsulated MN either in PNCs or LNCs showed higher cell viability in WISH amniotic cells in comparison with free MN. PNCs showed less *ex vivo* permeation. PNCs were accompanied by high stability and more amount drug deposition (32.2 ± 3.52 µg/cm^2^) than LNCs (12.7 ± 1.52 µg/cm^2^). The antifungal activity of the PNCs was high 19.07 mm compared to 11.4 mm for LNCs. In conclusion, PNCs may have an advantage over LNCs by offering dual action for both superficial and deep fungal infections.

## 1. Introduction

The incidence of skin fungal infections is growing nowadays affecting millions of people every year worldwide (Sousa et al., 2020). Superficial fungal infections, commonly known to target skin, hair, nails, and mucosal tissues, are mostly caused by Cryptococcus, Candida, Aspergillus, and Pneumocystis. They are more frequent and life threatening than systemic fungal infections (Gupta et al., 2017; Bolla et al., 2019). They are more observable in patients suffering from immunosuppressing diseases like AIDS, tuberculosis, cancer, and chronic obstructive pulmonary disease (Bolla et al., 2019). Recently, fungal infections were highly observed in patients suffering from severe COVID-19 symptoms followed by post COVID 19 syndrome. The most common fungal infections in patients with COVID-19 include aspergillosis or invasive candidiasis (Hoenigl, 2020; El-Kholy et al., 2021). About half of the patients suffering from immune-compromised issues are susceptible to be infected with *Candida albicans*, the most abundant fungal pathogen affecting humans (Maródi & Johnston, 2007). Symptoms of candidiasis are various ranging from severe redness, swelling, itching resulting in skin fissures or sores, white patches to pelvic pain and bloody urine according to site of infection (Mayer et al., 2013). Topical anti-fungal drug delivery is looked up to as the most preferable pathway in treatment of major superficial skin fungal infections, ensuring its direct access and higher retention rate at the target (Garg et al., 2020). Topical delivery further subsidizes limitation of pre-systemic metabolism of the drug to enhance bioavailability. Moreover, it increases the efficacy of treatment, allows potential self-medication, and increased patient compliance (Bolla et al., 2019). However, topical preparations showing poor skin penetration caused adverse skin reactions such as skin irritation, allergic reactions, and itching (Kulawik-Pióro & Miastkowska, 2021). They also showed variable drug levels at the site of infection with unfortunate dermal and ungual bioavailability. Therefore, novel drug delivery system is envisaged to address the problems associated with topical preparations reducing local side effects and increasing their therapeutic efficacy. Some of these novel carriers are liposomes, niosomes, solid lipid nanoparticles (SLNs), nano-lipid carriers, and polymeric nanoparticles which highly evolve the advent of targeted delivery and drug stability (Verma & Utreja, 2019; Montoto et al., 2020).

Miconazole nitrate (MN) is a broad-spectrum antifungal drug of the azole derivatives extensively used for the treatment of dermatophytosis, cutaneous mycosis, and fungal infections affecting the vagina, mouth and skin, including candidiasis (Aljaeid & Hosny, 2016). Miconazole is a weak base (pKa = 6.7) with poor aqueous solubility (1.03 µg/mL) and rapid clearance hindered its systemic efficacy (Jain et al., 2010). MN acts by dual pathways: impedes the synthesis of ergosterol on fungal cell and causes accretion of reactive oxygen species (ROS) in the fungal cell, triggering oxidative damage, and cell death (Amaral et al., 2019). Currently, miconazole is available as conventional topical formulations such as cream, lotions, spray liquids, and suppository for vaginal use. Previously published reports stated that miconazole topical applications exhibited poor skin penetration and therefore higher doses are required to recompense low permeability (Qushawy et al., 2018). Moreover, it suffers from vast side effects on the application site like burning, redness, and swelling (Kenechukwu et al., 2018). Therefore, various approaches have been attempted to overcome these hitches and consequently improve the therapeutic efficacy of drugs such as SLNs (Aljaeid & Hosny, 2016), nano-suspensions (Cerdeira et al., 2010), transdermal films (Ofokansi et al., 2015), ethosomes, liposomes, and nanostructured lipid carriers (NLCs) (Firooz et al., 2016).

Recently, nanocapsules attracted more interest in drug delivery applications, profiting from their core–shell nanostructure. Nanocapsules are distinctive class of nanoparticles, nanosized carrier composed of two main parts (core) which is oily in nature and a protective thin polymer matrix (shell) constrained therapeutic substance (Nilewar et al., 2017). They are fruitfully encapsulating both hydrophilic and lipophilic drugs according to their natures with high drug loading (%DL) capacity. Moreover, they enhanced drug solubility, controlled drug release declining burst release induced by pH, temperature, enzymes, and other factors and improve drug stability (Deng et al., 2020). Additionally, they reported to improve the efficacy of candidiasis pharmacological treatment. Nanocapsules were classified into polymeric nanocapsules (PNCs) and lipid nanocapsules (LNCs), which are different in their compositions. PNCs are composed of a core enclosed by drugs inside and enfolded by polymeric membrane (Nagaich, 2018), while LNCs are hybrid between both liposomes and nano-emulsions which are composed of a liquid, oily core (medium-chain triglycerides) enclosed by both hydrophilic and lipophilic surfactants (Saliou et al., 2013). Both are used for the delivery of peptides, proteins, genes, and several compounds. The most commonly used polymers for forming the shell are biocompatible and biodegradable such as poly lactic acid, polylactide-co-glycolide, and poly (Ɛ-caprolactone). Polycaprolactone (PCL) is widely used to prepare different types of nanocapsules comparing to other biodegradable polymers owing to its talented characters; safety, elasticity, cytocompatibility, and very slow biodegradation which were approved by US-FDA (El-Hesaisy & Swidan, 2020; Rahat et al., 2021). It is also useful to capture a wide range of drugs such as anti-cancer drugs, anti-inflammatories, and immune modulators presenting diverse flexibility with potential applications in therapy (Mahareek et al., 2019). PCL nanocapsules are malleable formulations used in the liquid form as well as can be incorporated into semi-solid or solid dosage forms. In LNCs, medium and long chain fatty acids are commonly used such as propylene glycol (PG) dicaprylocaprate (Labrafac^®^) and oleic acids (Pohlmann et al., 2013). They are synthesized by nanoprecipitation, emulsification, solvent displacement, and solvent evaporation techniques (Govender et al., 2015). Among these methods, emulsification technique was elected in this study to prepare MN loaded nanocapsules as it is simple, inexpensive, and most widely used method in preparing nanocapsules suspension system (Deng et al., 2020).

Our study aimed to prepare and evaluate both polymeric and LNCs for topical delivery of MN to provide an innovative way to enhance its antifungal activity with minimal side effects, reducing the dose and dosing frequency associated with other conventional topical drug delivery system. Developed nanocapsules were characterized with respect to particle size, drug entrapment, surface morphology, *in vitro* diffusion and stability studies, and *ex vivo* retention and permeation. The effect on cell viability and antifungal activity was also investigated.

## 2. Materials and methods

### 2.1. Materials

Miconazole nitrate was kindly donated from Sedico Pharmaceutical Company (6th of October City, Egypt). Polycaprolactone was purchased from Sigma-Aldrich Chemical Co. (St. Louis, MO). Propylene glycol dicaprylocaprate (Labrafac^®^) was given as a gift by Gattefosse (Saint-Priest, France). Sodium dihydrogen phosphate and disodium hydrogen phosphate were obtained from El-Gomhouria Chemicals Pharmaceutical Company (Cairo, Egypt). Propylene glycol, dichloromethane, and Tween 80 were all pharmaceutical grades and were attained from El-Nasr Chemicals Pharmaceuticals Company (Cairo, Egypt). WISH cell line (normal human epithelial amniotic cells) was obtained at National Cancer Institute (NCI) (Cairo, Egypt). RPMI1640 nutritional media was purchased from Lonza Bioscience (Biological Products Company) (Morristown, NJ).

### 2.2. Preparation of MN loaded polymeric nanocapsules

Polymeric nanocapsules (PNCs) were prepared by emulsification/nanoprecipitation method with limited modifications (Xia et al., 2017; Jahangir et al., 2018; Deng et al., 2020; Oliveira et al., 2020). It is the most simple and cost effective method. PCL biodegradable polymer is employed in PNCs. The organic and aqueous phases were prepared separately. The organic phase containing MN and poly (ε-caprolactone) (PCL) dissolved in 10 mL dichloromethane at ratios 1:2, 1:3, and 1:4 under vigorous stirring (1200 rpm) at room temperature using magnetic stirrer for 15 min (Table 1).

**Table 1.** Composition of different prepared MN loaded polymeric and lipid nanocapsules.

Formula code	MN (mg)	MN:PCL	MN:lipid matrix	Surfactant type (w/v)		Nanocapsules type
				Tween 80 (%)	Soy phosphatidylcholine (g)	
F1	20	1:2	–	5%	–	PNCs
F2	20	1:3	–	5%	–	PNCs
F3	20	1:4	–	5%	–	PNCs
F4	20	–	1:2	–	5	LNCs
F5	20	–	1:3	–	5	LNCs
F6	20	–	1:4	–	5	LNCs

Meanwhile, the aqueous phase was composed of aqueous solution of Tween 80 (5% w/v) and 10% glycerol (based on preformulation studies to screen the effect of surfactant type) (Jahangir et al., 2018). The aqueous phase was added dropwise using syringe to the previous organic phase and the mixture was stirred at 2000 rpm for 30 min at room temperature to obtain the required PNCs suspension. The prepared PNCs were centrifuged at 4000 rpm for 15 min and the obtained pellets were re-dispersed again in de-ionized water. Centrifugation cycle was repeated three times to get rid of organic solvents that have to be strictly eliminated from the formulations. The effect different ratios of MN:PCL (1:2, 1:3, and 1:4) on PS, ZP, and %EE were further studied.

### 2.3. Preparation of MN loaded lipid nanocapsules

Based on preformulation studies (data unseen) and previous reports, MN loaded LNCs were prepared using lipid matrix of both oleic acid and Labrafac^®^ oil with ratio 1:1 (Kamel & Basha, 2013; Eissa et al., 2015; Kiani et al., 2017). The previously mentioned oil mixture has shown high solubilization power for MN referred to the long chain of oleic acid and the HLB value. Briefly, 20 mg of MN was mixed with lipid matrix (melted in water bath at 80 °C) at different ratios (1:2, 1:3, and 1:4) where soy phosphatidylcholine was used as surfactant (Table 1). A hot aqueous solution was prepared by dissolving soy phosphatidylcholine (5 g) in PG (10 g) in a mass ratio of 1:2. The aqueous solution was added using syringe to the previous lipid phase and the mixture was stirred at 1000 rpm for 15 min at room temperature to obtain the required nanocapsules suspension. The obtained LNCs suspensions were ultrasonicated for 5 min using probe sonicator (UP50H, Hielscher, Teltow, Germany). The composition of prepared polymeric and LNCs formulations is shown in Table 1. The effect of different ratios of MN:lipid matrix (1:2, 1:3, and 1:4) on PS, ZP, and %EE was further studied.

### 2.4. Characterization of developed PNCs and LNCs

#### 2.4.1. Particle size, polydispersity index, and zeta potential

The average particle size, polydispersity, and zeta potential of the MN loaded nanocapsules were determined using dynamic light scattering integrated in a zeta-sizer Nano-ZS (Malvern Instruments Ltd., Worcestershire, UK). Five milligrams of samples were diluted with a fixed amount of de-ionized water (10 mL) to obtain a suitable scattering intensity, filtered using a 0.22 μm filter (Millipore Co., Billerica, MA) and placed into disposal cuvettes (size). Three measurements were performed for each sample at an angle of 90° at room temperature (25 °C) using two refractive indexes (1.46 and 1.63) for polymer PCL and MN, respectively. Polydispersity index (PDI) was determined for assessing the particle size distribution and the homogeneity of the nanocapsules. Zeta potential was also determined to confirm the stability of the nanocapsules (Danaei et al., 2018).

#### 2.4.2. Determination of the drug loading and entrapment efficiency (%EE)

Drug loading and %EE of MN in the prepared nanocapsules were determined indirectly (El-Leithy & Abdel-Rashid, 2017). The concentration of free MN was measured in aqueous supernatant solution after separation of nanocapsules by centrifugation for 20 min at 10,000 rpm at 4 °C in high-speed cooling centrifuge (XCHR20, Bio Lion, Shanghai, China). The supernatant was filtrated through 0.22 µm membrane filter and the amount of MN entrapped was detected spectrophotometrically (Perkin Elmer UV, Yokohama, Japan) at *λ*_max_ 280 nm after suitable dilution with methanol. Each experiment was carried out in triplicate and the mean value was deduced. The %DL and %EE were calculated by the following equations:

$$\text{\% Drug loading content }=\;\frac{\text{weight of the drug in NCs}}{\text{weight of the NCs}}\;\times \;100$$

$$\text{\% Encapsulation efficiency }=\;\frac{\text{initial amount of MN added to NCs}-\text{free MN in supernatant}}{\text{ initial amount of MN added }}\times \;100$$

### 2.5. Transmission electron microscopy (TEM)

The shape and outlines of prepared NCs were inspected using TEM (Jeol, JEM, Tokyo, Japan). Freshly prepared samples (diluted appropriately with 0.1 M phosphate buffer) were deposited onto the surface of carbon coated copper grids; natively stained with 2% phosphotungstic acid and dried at room temperature. The stained sample was then probed and visualized using TEM (Mora-Huertas et al., 2010).

### 2.6. *In vitro* release study for prepared MN nanocapsules

The release of MN from PNCs and LNCs formulations was investigated in phosphate buffer (pH = 7.4) solution using dialysis bag (regular dialysis) method (Govender et al., 2015; Dar et al., 2018). The dialysis bag (molecular weight cut off: 12–14 kDa, Livingstone, Sydney, Australia) was first soaked in phosphate-buffered saline (PBS) at pH 7.4 overnight before use. Nanocapsules suspension of selected optimized formulations equivalent to 50 µg of MN was placed inside the dialysis bag, tied at both ends and immersed in 100 mL of PBS (pH 7.4, 37 °C) release medium containing 0.25% sodium lauryl sulfate. The sinking conditions were taken into consideration.

The solution was stirred at 100 rpm with the help of the magnetic stirrer at 37 ± 0.5 °C. At scheduled time intervals, 2 mL of the release media was removed, filtered through a 0.22 μm Cameo Acetate membrane filter (Millipore Co., Billerica, MA) and replaced by fresh release medium. The withdrawn samples were analyzed spectrophotometrically (Perkin Elmer UV, Yokohama, Japan) at *λ*_max_ 280 nm for MN content. For the sake of comparison, release pattern of 5 mg free MN suspension from dialysis bag was also conducted at the same conditions. The release data were subsequently fitted to different release kinetic models representing (zero-order, first-order, Higuchi diffusion, Hixon and Baker equations) to determine the release kinetics. Correlation coefficient (*R*^2^) values were compared for selection of the most appropriate release model that best fits the data (Nasr et al., 2020).

### 2.7. Effect of storage on stability of prepared nanocapsules

The stability of optimum MN loaded nanocapsules suspensions was studied by storage of three samples in sealed vials at room temperature 25 °C for 3 months. During this period, %EE, PS, PDI, and ZP of the nanocapsules were measured as described previously. Nanocapsules were examined visually for aggregation and change in their appearance. Statistical significance was analyzed by Student’s *t*-test using SPSS^®^ software 22.0 (SPSS, Chicago, IL). Difference at *p*>.05 was considered insignificant.

### 2.8. Antifungal potential of MN-loaded NCs

The antifungal effect of the optimized MN-loaded NCs was studied using diffusion agar method (Dudhipala & Ay, 2020). One milliliter of standard strain of *Candida albicans* ATCC 76615 (1 × 10^6^ CFU/mL) was cultivated, then inoculated in petri dishes of 150 mm diameter containing 50 mL of the Müller–Hinton agar. Holes of 10 mm diameter were made and filled with 100 µL containing 5 mg of drug suspension or the equivalent amount optimized MN-loaded NCs. The petri dishes were incubated for 4 h at 37 °C. The area where there is disappearance of fungal growth around the holes (inhibition zone) was measured using a caliper. For a full view, the antifungal activity of a positive control (free MN suspension) was implemented (Ahmed & Aljaeid, 2017).

### 2.9. Effect of nanocapsules on cell viability (cytotoxicity and genotoxicity)

#### 2.9.1. Cell culture and treatment

Remembering that cell culture is a mirror environment for the bio-internal one, the WISH cell line (normal human epithelial amniotic cells) was kept at 37 °C under 5% CO_2_ using a water jacketed carbon dioxide incubator. In 96-well microliter plastic plates at concentration of 10 × 10^3^ cells/well, the cells were cultivated for five days at sterile area using a laminar flow cabinet biosafety class II level in a specific nutritional medium (RPMI 1640), with 1% antibiotic–antimycotic mixture supplement (10,000 µg/mL potassium penicillin, 10,000 µg/mL streptomycin sulfate, and 25 µg/mL amphotericin B), 10% fetal bovine serum and 1% l-glutamine (El-Leithy et al., 2019). The media of different plates were aspirated and replaced with fresh medium. The WISH cells incubated in fresh medium were taken as a negative control. However, other cell line plates were treated with free MN, and the equivalent weights of selected nanocapsules formulations. The samples were prepared to reach various concentrations of drugs (0.25–100 µg/mL).

#### 2.9.2. Determination of cell viability (cytotoxicity)

The effect of free and encapsulated MN on viability of WISH amniotic cells was studied using MTT assay (Qi et al., 2009). MTT salt (2.5 μg/mL) was added to each well to be incubated for 4 h at 37 °C under 5% CO_2_. Unbound MTT and dead cells found in each well were removed by suction and subsequently exchanged with 200 μL of 10% sodium dodecyl sulfate. All experimental assays were carried out in triplicate. A cytotoxic natural agent that gives 100% lethality positive control was used as positive control under the same conditions. The plates were then read at *λ*_max_ 595 nm using a microplate multi-well reader. The percentage of change in viability was calculated according to the formula:

Viability %=(optical density of sample/optical density of control) × 100

A probit analysis was conducted to determine LC50 using SPSS 11 program (SPSS, Chicago, IL) and the microplates were photographed using inverted microscope. The LC50 is the lethal concentration of the sample which causes the death of 50% of cells in 48 h.

#### 2.9.3. Comet assay (genotoxicity)

Comparing to other assays, the comet response in detecting DNA damages was elected as it was the more sensitive, rapid, and reproducible assay (Gunasekarana et al., 2015). Comet assay analysis was implicated to investigate the effect on DNA of WISH amniotic cells which may give an indication for death pathway. Trevigen's Comet Assay^®^ kit (Trevigen, Inc., Gaithersburg, MD) and Comet Image Analysis System software (Comet Scores software; TriTek, Sumerduck, VA) were used to analyze; tail length, % of DNA in the tail, and tail moment.

### 2.10. *Ex vivo* skin permeation and retentivity

This study evaluated the ability of PNCs and LNCs as drug delivery systems to enhance topical permeation/retention of MN (Jain et al., 2010; Nasr et al., 2020). The protocol of the study was approved by the Animal Ethics Committee of Faculty of Pharmacy, Helwan University. The experiment was conducted using excised full thickness dehaired abdominal rat skin (male albino rats, Sprague-Dawley; 100 g). The skin sections were installed on modified Franz diffusion cells (Crown Glass Co., Somerville, NJ) with an available permeability surface of 1.76 cm^2^ and a receptor volume of 50 mL such that the dermal side of the skin faced to the receptor fluid (PBS; pH 7.4). One milliliter of optimized MN-loaded NCs formulas (equivalent to 5 mg MN) were placed in the donor compartment at 37 °C. The sampling was performed at various intervals up to 24 h, where MN content was estimated spectrophotometrically at 280 nm (Salah et al., 2017). Subsequently, the skin was removed and washed 10 times with a cotton swab followed by weighing and homogenizing in methanol. The produced solution was centrifuged for 10 min at 5000 rpm and supernatant was filtered then analyzed for drug amount spectrophotometrically to determine percentage drug skin deposition (Nasr et al., 2020). A similar study was also performed for free MN suspension for sake of comparison. The study was carried out in triplicate for both.

### 2.11. Statistical analysis

Data inspection was accomplished using GraphPad InStat 3 program (GraphPad Software, La Jolla, CA). Results were stated as a mean ± standard deviation. Statistically significant difference was determined using one-way ANOVA test and paired and un-paired Student’s *t*-test with *p*<.05 as a minimal level of significance.

## 3. Results and discussion

### 3.1. Particle size, PDI, and zeta potential

The results of particle size, PDI, and ZP of the prepared nanocapsules are displayed in Table 2. The measured particle size was in the nanometric size range, which is preferred for drug delivery and penetrability to biological cell (Subramaniam et al., 2020). The average particle size of PNCs suspensions (F1, F2, and F3) ranged from 108 ± 3.63 nm and 180 ± 2.11 nm. The results obviously showed a statistically significant directly proportional function between drug–polymer ratio (MN:PCL) and average particle size of nanocapsules suspensions at constant surfactant concentration Tween 80 (5% w/v). These results were in line with findings of Sathyamoorthy et al. (2017); who reported that the particle size increases by increasing PCL concentration which results in increased incidence of collisions between particles during emulsification consequently aggregation of small sized particles yielded large particle size. Moreover, the increase of PCL concentration increases viscosity and resistance of the organic phase to diffuse and distribute into the aqueous phase that prompts the formation of large nanodroplets at the interface (Ajiboye et al., 2018). Additionally, the high viscosity could decline the drug diffusion from the nanocapsules as another cause for increasing particle size (Tavares et al., 2017). Tween 80 (HLB 15) was chosen as a surfactant after preliminary studies compared to span 60 (4.7) (data unseen). It was found that hydrophilic surfactants strongly participate in decreasing particle size of nanocapsules by causing high droplets stabilization and more flexibility (Zhu et al., 2005). Furthermore, the zeta potential of PNCs suspensions (F1, F2, and F3) was −31 ± 3.10, −35.03 ± 4.80, and −40 ± 5.21, respectively. PCL is known for yielding spherical nanocapsules with negative charge on the surface (Rahat et al., 2021). They displayed negative charges owing to PCL structure and its hydrophobic nature which may cause ionization of the carboxylic groups resulting in a negative potential to the interface (Michels et al., 2019; Lino et al., 2020). Therefore, increased concentration of PCL influences negativity of zeta potential of prepared nanocapsules. The high negative zeta potential owing to the charges repulsion toward the natural tendency of aggregation affords stability to nanocapsules from the hostile pH of the biological system (Alves et al., 2016; Rahat et al., 2021).

**Table 2.** Characterization of prepared MN nanocapsules.

Formula code	DL (%)	EE (%)	Particle size (nm)	PDI	Zeta potential (mV)
F1	20.1 ± 0.80	83 ± 3.10	108 ± 3.63	0.33	–31 ± 3.10
F2	23.1 ± 0.65	87 ± 4.80	120 ± 1.63	0.35	–35.03 ± 4.80
F3	25.7 ± 0.79	91 ± 3.72	180 ± 2.11	0.4	–40 ± 5.21
F4	19.2 ± 0.72	80 ± 5.21	116 ± 1.63	0.21	–25 ± 3.72
F5	20.1 ± 0.60	88 ± 3.72	95 ± 2.11	0.31	–29.23 ± 1.2
F6	24.1 ± 0.65	98 ± 5.21	89 ± 3.63	0.20	–31.22 ± 2.1

On the other hand, the results displayed a negative correlation between MN:lipid ratio and average particle size of LNCs at constant soy phosphatidylcholine concentration (5 g) with values ranging between 89 ± 3.63 and 116 ± 1.63 nm. By increasing lipid content, the average particle size decreased; this could be revealed to the combination of labrafac and oleic acid which attained good solubilization for MN and proper self-emulsification yielded small droplet size (Kamel & Basha, 2013). Previously, published reports stated that short chain fatty acid ester of labrafac attained small droplets (Atef & Belmonte, 2008). Moreover, oleic acid can reduce the interfacial tension creating smaller and smoother particles (Sanad et al., 2010). Furthermore, the high phosphatidylcholine concentration was reported to produce smaller particles by stabilizing the system formation more efficiently (Kassem et al., 2021). The results also showed that LNCs exhibited relatively low average particle size compared to PNCs referred to low viscosity of the lipid melt unlike PCL dispersion (Table 2).

The negative zeta potential of LNCs (–25.23 ± 3.72 to −33.22 ± 2.1) may be attributed to the amphiphilic surfactant soy phosphatidylcholine as well as presence of oleic acid in the oil core which has strong negative charge (Xia et al., 2017; El-Hesaisy & Swidan, 2020). Consequently, by increasing lipid content with respect to MN, zeta potential increased (Table 2).

The fabricated nanocapsules had PDI values extended from 0.2 to 0.35 indicating narrow size distribution, excellent sample size homogeneity and reproducible method of preparation which was suitable for topical delivery applications (Xia et al., 2017).

### 3.2. Entrapment efficiency of prepared MN nanocapsules

The effect of the MN:PCL ratio and MN:lipid ratio on the %EE of MN in prepared nanocapsules is displayed in Table 2. The %EE was in the range between 80 ± 5.21 and 98 ± 5.21. Generally, the high %EE of MN may be attributed to its high lipophilic nature (log *P* 5.96). Increasing PCL concentration with respect to MN had a considerable effect on %EE, by increasing its concentration the %EE increased (Rahat et al., 2021). The relatively high EE value was owing to the high chemotactic between MN and PCL as both are highly hydrophobic; hence, both entities have great affinity toward each other (Govender et al., 2015). Moreover, by increasing PCL concentration, the viscosity of primary emulsion formed increased hindering the distribution of MN into the external phase and consequently increases the entrapment of the drug (Tinca et al., 2020).

The results in Table 2 also revealed that the %EE values for LNCs (F4, F5, and F6) were higher than PNCs (F1, F2, and F3) and this could be related to the inclusion of lipids in the core of nanocapsules structure where highly lipophilic drugs could be concentrated in (El-Hesaisy & Swidan, 2020). As well as they donate thick shell for nanocapsules; allow higher amount of drug entrapment as well as slower release (Radwan et al., 2020). Moreover, oleic acid and Labrafac^®^ (medium chain fatty acid ester) were reported previously as good solubilizers for lipophilic drugs profiting high %DL (Kamel & Basha, 2013). Therefore, by increasing lipid contents the %EE increased, F6 > F5 > F4. Based on the previous results, PS, zeta potential, %DL, and %EE (Table 2), F1 and F6 showed insignificance; therefore, they were subjected for further studies.

### 3.3. Selection of best formula

Based on the previous results, it was concluded that the formula coded F6 could be selected as optimum LNCs formula. It showed small particle size, PDI, and ZP; 89 nm, 0.2 and −31.22, respectively that synchronized with high %EE (98%) (Table 2). This could be attributed to LNCs structural features which can entrap lipophilic drugs in their oil core that provide high space capacity for drug encapsulation and barrier for drug diffusion. Meanwhile, formula coded F1 was selected as optimum PNCs formula as it showed least particle size and PDI accompanied with high zeta potential and best %EE as shown in Table 2. For further comparison, both formulations were subjected to subsequent investigations.

### 3.4. Transmission electron microscope

**Figure 1.**
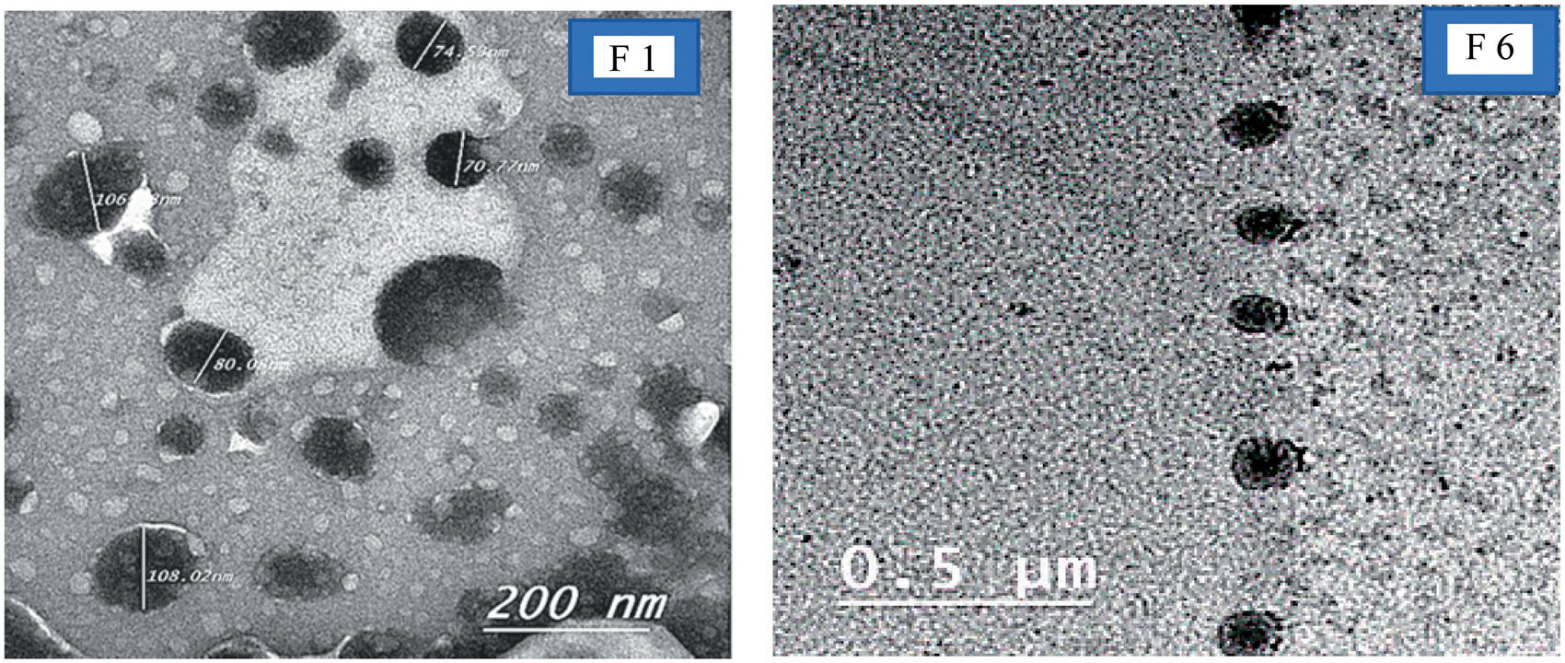
TEM images of optimized nanocapsules (F1, F6).

A representative TEM photomicrograph was conducted for F1 (optimum PNCs) and F6 (optimum LNCs) as illustrated in Figure 1. For F1, the photos showed small elliptical particles. The PNCs were light in color referred to poor absorption of phosphotungstic acid stain compared to LNCs (Abbas et al., 2015). The TEM examination showed spherical particles displaying a core–shell structure. It also revealed that the optimized formula (F6) had an almost homogeneous small-sized spherical appearance with a narrow size distribution and not aggregated. The particle size of nanocapsules appears to match the results obtained previously by zeta sizer.

### 3.5. *In vitro* release study

**Figure 2.**
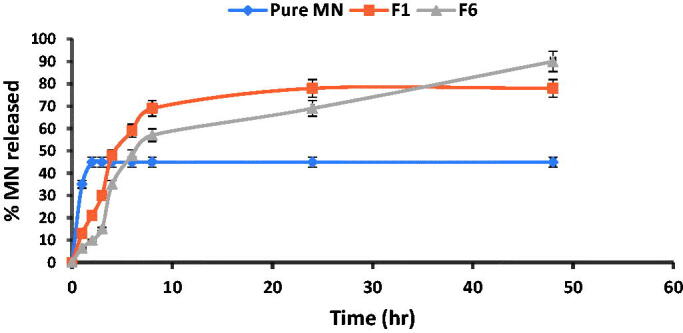
*In vitro* release profiles of miconazole nitrate solution (MN), miconazole-loaded PNCs (F1) and miconazole-loaded LNCs (F6) formulas in phosphate-buffered saline (pH = 7.4) at 37 °C ± 0.5 °C.

The *in vitro* release profile is a mirror image for predicting *in vivo* drug performance. The release profiles of MN from prepared PNCs (F1) and LNCs (F6) are illustrated in Figure 1. The pure drug revealed a quicker release within the first two hours (45%), followed by a plateau pattern. However, encapsulated MN showed slow release from PNCs and LNCs formulations over a period up to 48 h. From the results, both PNCs (F1) and LNCs (F6) showed a biphasic drug release profile; displayed an initial rapid % drug release of 30 ± 2.4 and 15 ± 1.9, respectively, in the first 3 h. The statistically significant difference in amount of drug released from PNCs and LNCs in the starting 3 h could be explained by the difference in the structural composition of the two types of nanocapsules. The polymeric type (F1) of nanocapsules showed nearly two folds burst effect compared to the lipid type (F6) owing to deposition of some of the loaded amount of MN on the surface of PNCs (shell). Whereas, the LNCs may have offered more room for %DL in the core part due to the oily nature of LNCs core (Dubey et al., 2020; Lino et al., 2020). Another reason, suggested for the previously resulted release pattern of PNCs is the degradation of the thin polymeric outer shell membrane (Cauchetier et al., 2003). The initial burst effect was followed by more controlled MN release reaching 78 and 90%, after 48 h. The controlled release behavior attained by PNCs (F1) was correlated to the slow diffusion of MN from the viscous PCL matrix as a result of its degradation which is considered as an obstacle for its distribution into the external dissolution medium (Midhun et al., 2011). However, the low concentration of PCL with respect to MN in F1 makes the release of MN more rapid and completed within 24 h with maximum amount released 78%. On contrast, the release of MN from LNCs (F6) was controlled and extended to 48 h with maximum amount released 90% (Figure 2). This finding was attributed to the high lipophilicity of MN as well as the structural composition of lipid core matrix (high lipid content 1:4) and their high chemotactic which enclosed drug deeply and thus delays its diffusion (Kiani et al., 2017). Moreover, the rigid external shell of LNCs presents an obstacle for drug diffusion from oil core to the external phase; therefore, it is less probable at the nanocapsules surface. Consequently, LNCs exhibited sustained release functions more than PNCs.

Results of cumulative drug diffused were subjected to release kinetics models. The results portrayed that free MN, F1, and F6 release have followed zero order, Higuchi diffusion and Baker and Lonsdale models, respectively, based on regression coefficient values (Table 3). Pure MN displayed a continuous release of the same amount of drug per unit time (zero order) which correlated to its lipophilicity that is characterized by release kinetics of this nature (Rodrigues et al., 2020). In turn, PNCs (F1) are best fitted to Higuchi’s diffusion model where the drug released from the nanocapsules after the degradation of thin polymeric outer shell membrane is governed by the slow diffusion of MN from the viscous PCL matrix rather than erosion mechanism (El-Hesaisy & Swidan, 2020). Moreover, the LNCs (F6) were fixed to the Baker and Lonsdale model which designates controlled drug release manner from spherical-core shell matrices combined with diffusion (Mircioiu et al., 2019). This result was agreed with rigid external shell of LNCs and MN high lipophilicity which delay drug diffusion from oil core to the external phase and hence achieving controlled drug release mechanism.

**Table 3.** Kinetics analysis of the *in vitro* release data from MN-loaded nanocapsules formulations (F1 and F6) comparing to pure MN.

Formulations code		Correlation coefficients (*R*^2^)				Order of reaction
	Zero order	First order	Higuchi diffusion	Hixon	Baker and Lonsdale	
Pure MN	0.521	0.242	0.305	0.313	0.341	Zero order
F1	0.717	–0.057	0.869	0.698	0.834	Higuchi diffusion
F6	0.861	–0.223	0.951	0.91	0.987	Baker

### 3.6. Stability study

**Figure 3.**
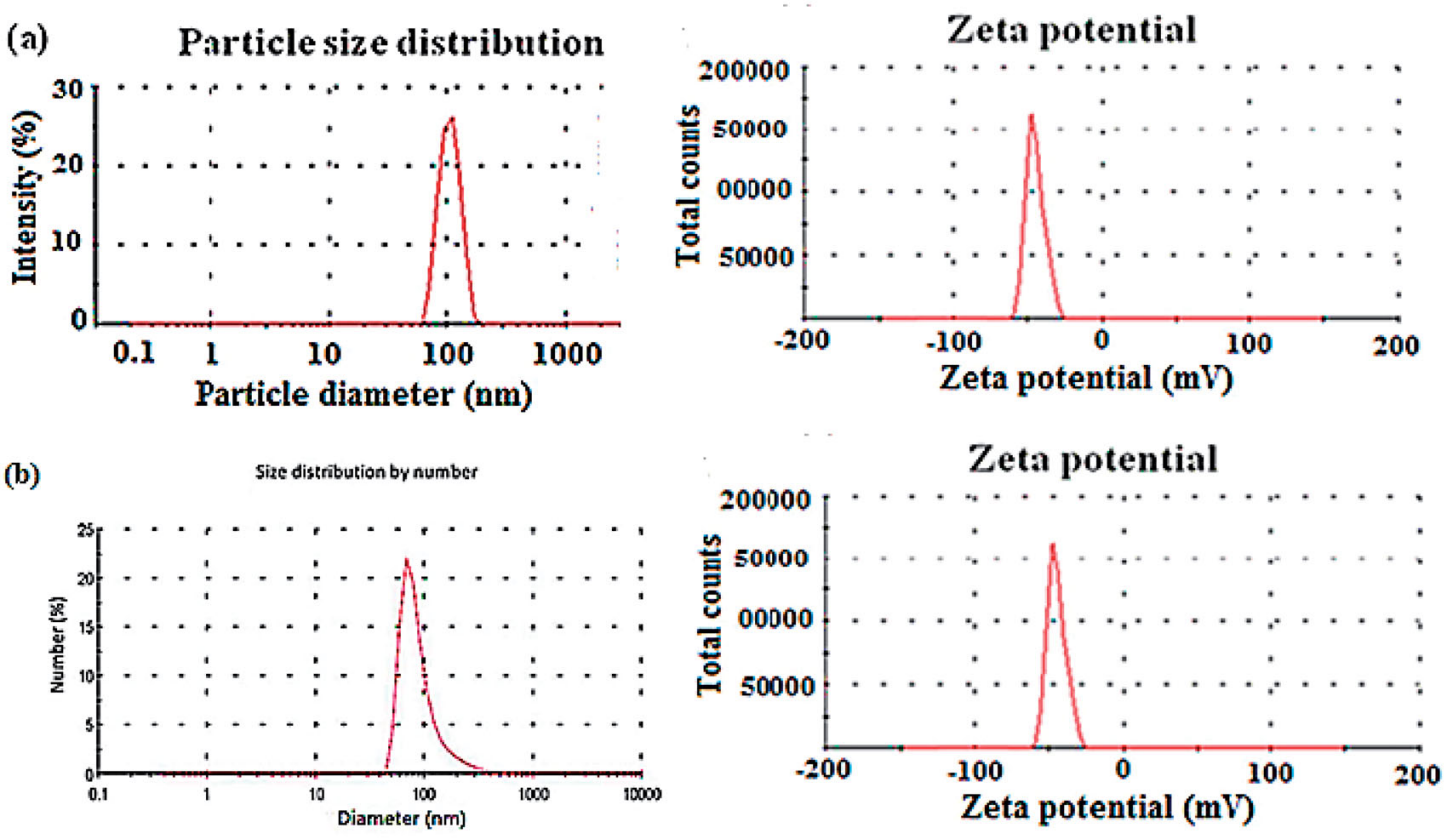
Particle size distribution (nm) and zeta potential (mV) of stability data for (a) MN loaded in PNCs (F1) and (b) MN loaded in LNCs (F6).

After 3 months of storage at 25 °C, the selected formulas (F1 and F6) kept its physicochemical properties (Table 4, Figure 3). The results were found to be statistically insignificant (*p*>.05, paired *t*-test) with those obtained before storage, indicating the stability of MN-loaded nanocapsules. However, the PNCs (F1) seem to be more stable than LNCs (F6) during and after storage owing to the presence of PCL with regard to synthetic and biodegradable polymer keeping stability, while LNCs structural features and their oil core tend to aggregation and deformation (Deng et al., 2020).

**Table 4.** Effect of environmental storage on %EE, particle size, and zeta potential of selected MN-loaded nanocapsules formulas (F1 and F6).

Parameters	Storage periods (months) at 25 ± 2 °C			
	F1		F6	
	0	3	0	3
%EE	83 ± 3.10	81 ± 3.21	98.12 ± 5.21	90.6 ± 3.21
Particle size (nm)	108 ± 3.63	106 ± 1.25	89 ± 3.63	97 ± 3.5
PDI	0.33	0.35	0.2	0.3
Zeta potential (mV)	–25 ± 3.10	–27 ± 2.8	–33.22 ± 2.1	–28 ± 3.21

### 3.7. Antifungal activity of MN-loaded nanocapsules

The results of antifungal activity study, presented as the inhibition zone diameter, were in direct correlation with the *in vitro* release data. The antifungal activity of the optimized PNCs (F1) was high 19.07 mm compared to 11.4 mm for optimum LNCs (F6) and only 5 mm with a drug suspension (positive control). These results highlighted the enhancement of antifungal activity produced by MN after loading into different types of nanocapsules. To study effect on the minimum inhibition concentration (MIC), different dilutions of tested samples were applied. It was found that nanocapsules as drug delivery system can improve the anti-fungal activity of MN by decreasing the needed MIC from 2 µg/mL to nearly 0.75 µg/mL (Ahmed et al., 2021).

### 3.8. Effect of nanocapsules on cell viability (cytotoxicity and genotoxicity)

**Figure 4.**
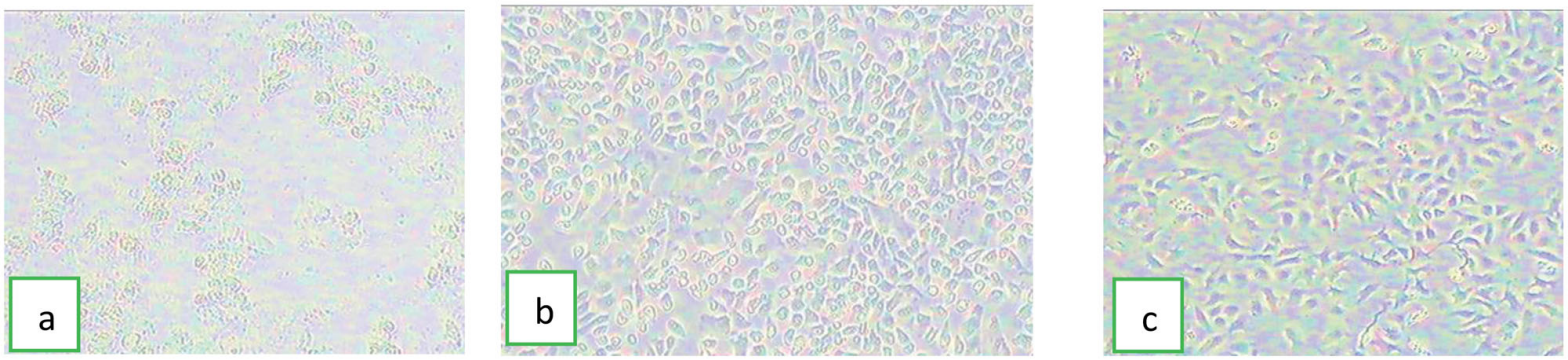
Optical images demonstrating the effect of (a) free MN, (b) MN loaded in PNCs (F1), and (c) MN loaded in LNCs (F6) on cell viability of WISH cell lines (magnification= ×400 inverted microscope).

The results of MTT assay revealed the low cytotoxicity of MN in either free or encapsulated forms (Table 5 and Figure 4). The optical images captured by inverted microscope showed samples treated by free MN that completely lost integrity of cells as a sign of cytotoxicity (Figure 4(a)). On the contrary, the optical images showed the ability of nanocapsules to keep cells in a healthy condition (Figure 4(b,c)). The results also indicated the effect of nanocarrier on cytotoxicity of MN, where LC50 of MN was increased from 45.4 µg/mL for free MN to 83.5 µg/mL and 89.2 for PNCs and LNCs, respectively. Based on a previously published report, iron oxide nanoparticles coated with chitosan nanocarrier did not reduce the cytotoxic potential compared with free MN (Caldeirão et al., 2021). This finding privileged the effect of using nanocapsules over other nanocarrier systems.

**Table 5.** % Cell viability exerted by free and encapsulated MN on WISH amniotic cell line.

Conc. (µg/mL)	%Cell viability		
	Free MN	PNCs (F1)	LNCs (F6)
100	35.5 ± 2.5	45.5 ± 1.5	49.9 ± 0.7
60	40.2 ± 0.7	57.5 ± 1.6	65.9 ± 0.7
40	58.2 ± 1.2	67.2 ± 1.9	80.2 ± 0.9
20	71.4 ± 4.0	81.2 ± 2.1	93.2 ± 4.3
10	82 ± 2.8	91.4 ± 4.1	100 ± 0.8
5	96 ± 0.9	100 ± 5.7	100.4 ± 5.1
2	100 ± 1.9	100 ± 2.2	100 ± 3.9
1	100 ± 0.1	100 ± 3.2	100 ± 0.6
0.5	100 ± 2.5	100 ± 1.7	100 ± 1.5
0.25	100 ± 0.5	100 ± 3.5	100 ± 0.9

**Figure 5.**
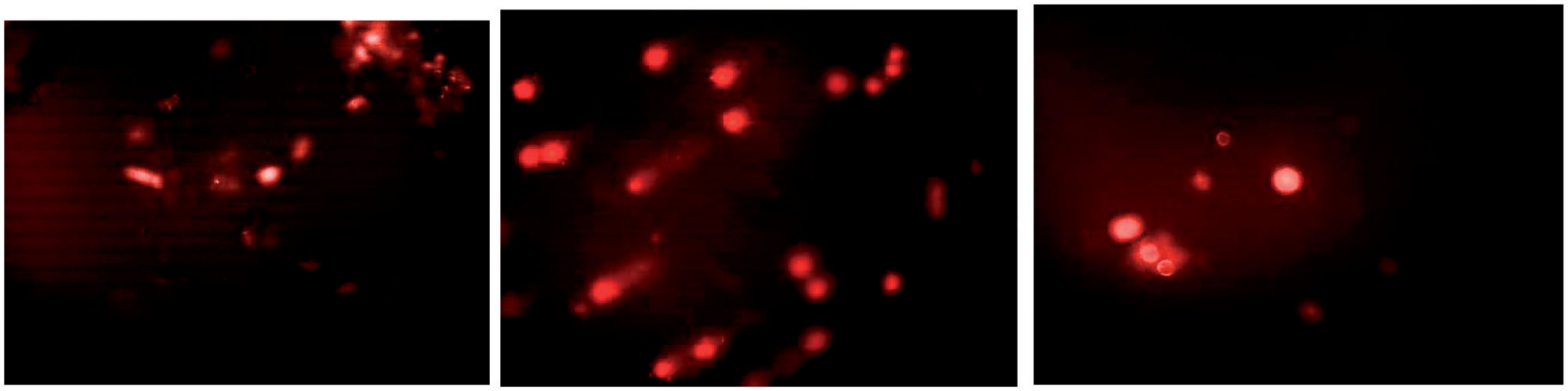
The comet assay images.

On studying effect on DNA damage, it was found out that there was slightly significant difference in tail moment of WISH amniotic cells for MN loaded in nanocapsules compared to free MN (1.2 ± 0.01 and 2.13 ± 0.07, respectively) (Figure 5). The cytotoxic effect of MN may be referred to the production of ROS in human keratinocytes, which may induce oxidative stress and cause cell death (Lam et al., 2020). Additionally, MN is known of causing inhibition in the growth of cells by activating extrinsic and intrinsic apoptotic pathways (Caldeirão et al., 2021). These findings, in conjunction with the antifungal activity results, indicate a synchronized advantage of MN loaded nanocapsules; lower cytotoxic potential and better antifungal effect.

### 3.9. *Ex vivo* skin permeation and retentivity

**Figure 6.**
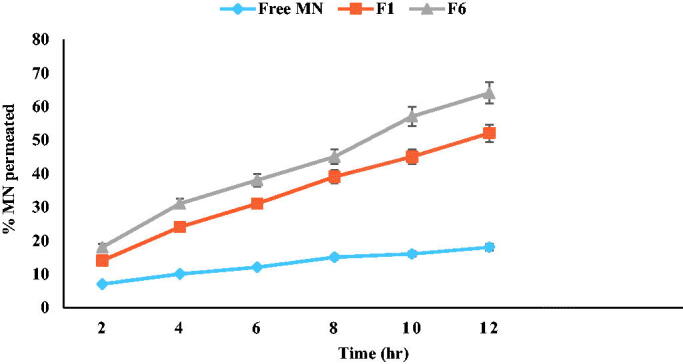
*Ex vivo* permeation profiles of MN from prepared miconazole-loaded PNCs (F1) and miconazole-loaded LNCs (F6) formulas compared to free MN suspension.

**Figure 7.**
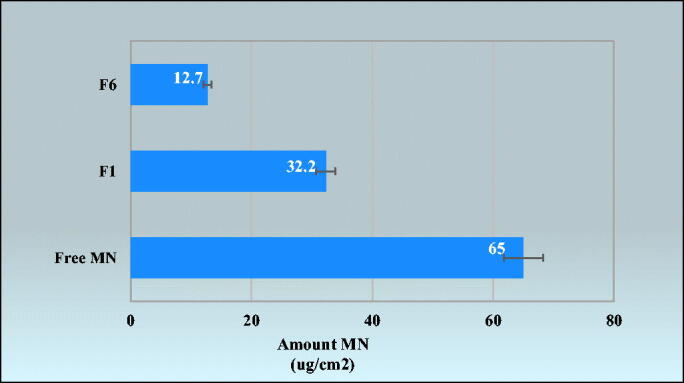
Amount of MN deposition on the skin from: free MN suspension, miconazole-loaded PNCs (F1) and miconazole-loaded LNCs (F6) formulas.

The results showed a very small amount of free MN could pass through skin samples reaching approximately 20% after 24 h. On the other hand, F1 (optimum PNCs) showed 71% permeability compared to 89% for F6 (LNCs) as observed in Figure 6. The results agreed with reports confirmed that only a negligible amount of MN could pass through skin (De Brum et al., 2015). It could be concluded that LNCs reached the dermis, while PNCs were more retained at the outermost layers of the skin. The result was in accordance with the flexibility in a way that higher flexibility gives deeper penetration and high compatibility of phosphatidylcholine in LNCs with skin composition (Coverdale et al., 2017; El-Leithy & Abdel-Rashid, 2017). Based on retentivity results as shown in Figure 7, it was found that F1 (PNCs optimum formula) showed more amount drug deposition compared to F6 (LNCs optimum formula). The results illustrated that free MN suspension; F1 and F6 were 65 ± 2.95 µg/cm^2^ and 32.2 ± 3.52 µg/cm^2^, and 12.7 ± 1.52 µg/cm^2^, respectively, after 24 h. Hence, the findings suggested that LNCs can reach the dermis and PNCs can act as reservoir systems at the epidermis (De Brum et al., 2015). Deposition of drug on the upper skin layers, reducing drug flux and creating a reservoir able to prolong skin residence time could provide better treatment to upper skin fungal infections (Peira et al., 2008). Moreover, PCL was used widely to control drug release owing to its high permeability in numerous pharmacological compounds as well as its extensive biocompatibility with living tissues and biodegradability through the hydrolytic rupture of ester bonds (Balcucho et al., 2020). The results confirmed that PNCs may have an advantage over LNCs by offering dual action for both superficial and deep fungal infections synchronized with biphasic release pattern.

## 4. Conclusions

MN loaded nanocapsules were successively prepared by simple and cost-effective technique; emulsification/nanoprecipitation using PCL biodegradable polymer in PNCs and lipid matrix (labrafac:oleic acid) at ratio 1:1 in LNCs. Both types of nanocapsules displayed small particle size, slow biphasic release manner, high %EE, and improved stability expressing a good approach for the delivery of MN. These findings, in conjunction with the antifungal activity results, indicate a synchronized advantage of MN loaded nanocapsules; lower cytotoxic potential and better antifungal effect. PNCs were more promising than LNCs offering dual action for both superficial and deep fungal infections synchronized with biphasic release pattern. Thus, PNC is an innovative way with promising results; can enhance antifungal activity with minimal side effects, reducing the dose and dosing frequency.
